# Knowledge and Attitudes regarding Temporomandibular Disorders among Postgraduate Dental Students and Practicing Dentists in Western China: A Questionnaire-Based Observational Investigation

**DOI:** 10.1155/2023/7886248

**Published:** 2023-07-18

**Authors:** Xin Xiong, Chuqiao Xiao, Xueman Zhou, Xiaojing Li, Jun Wang, Yating Yi

**Affiliations:** ^1^National Clinical Research Center for Oral Diseases, State Key Laboratory of Oral Diseases, Department of Orthodontics, West China Hospital of Stomatology, Sichuan University, Chengdu, Sichuan, China; ^2^National Clinical Research Center for Oral Diseases, State Key Laboratory of Oral Diseases, Department of Temporomandibular Joint, West China Hospital of Stomatology, Sichuan University, Chengdu, Sichuan, China; ^3^National Clinical Research Center for Oral Diseases, State Key Laboratory of Oral Diseases, Department of Head and Neck Oncology Surgery, West China Hospital of Stomatology, Sichuan University, Chengdu, Sichuan, China

## Abstract

**Background:**

It is necessary for dental students and dentists to apply their temporomandibular disorders (TMDs)-related knowledge to clinical practice. The current study aimed to evaluate the knowledge and awareness of postgraduate dental students and practicing dentists regarding etiology, diagnosis, and treatment of TMD in western China and thus provide suggestions on TMD curricula design to get postgraduate students and dentists better prepared for TMD diagnosis and treatment.

**Methods:**

This observational and descriptive cross-sectional study was conducted among postgraduate students and practicing dentists in western China. Twenty-five reorganized knowledge questions in four domains were selected from the published literature and were evaluated with answer options from “strongly agree” to “strongly disagree,” and “I don't know.” “Consensus” is defined as more than 50% of respondents in a group agree or disagree with a statement. Chi-square tests were performed for comparisons between the two groups.

**Results:**

A total of 132 postgraduate dental students and 123 dentists completed the questionnaire. Around 75% of postgraduate students and 85% of dentists claimed that they have never participated in systematic training in TMD. Nine statements in etiology, diagnosis, treatment, and prognosis of TMD had different consensus between the two groups. And the dentist group tended to agree more with 12 statements in the questionnaire.

**Conclusions:**

The majority of Chinese dentists and dental students have not taken any TMD courses and possess limited knowledge of TMD. Curriculum reform for predoctoral education, postgraduate education, and continuing education is needed to augment knowledge and skills for TMD diagnosis and treatment.

## 1. Introduction

Temporomandibular disorders (TMD) is a collective term used to define a number of clinical problems involving the masticatory muscles, temporomandibular joint (TMJ), or surrounding structures [1]. Common signs include restriction and deviation in jaw movements and muscle or TMJ pain during function and TMJ sounds. It affects women at the age of 20–40 years more frequently, with prevalence of around 30% in adults [2].

Despite of the multiple publications concerning TMD etiology and treatment, there is still no consensus on these topics [3]. This leads to the lack of confidence for dentists attending patients with TMD. When facing various related theories, they are likely to get confused and puzzled.

The multidimensional biopsychosocial model of TMD has been confirmed in a large number of fundamental studies. The biopsychosocial model indicates chronic pain, psychological distress, social factors, and mobility impairments should all be properly assessed in patients with TMD. The biopsychosocial model has been recommended by DC/TMD as one of the important principles of clinical considerations. However, it is found that some dentists have quite limited understanding of TMD and regard it as a simple somatic symptom or organic lesion in daily clinical practice, which often leads to underdiagnosis of patients, or giving improper advice on treatment options. Therefore, evaluating the current understanding of dentists on the diagnosis and treatment of TMD can help to develop appropriate educational strategies to enhance their level of TMD-related knowledge and awareness. So far, in order to understand knowledge and attitudes towards TMD, several studies have been carried out among either dental students or practicing dentists in several countries [4–6]. Conclusions are various in different areas based on different participants in each study. And attitude and knowledge towards TMD diagnosis and treatment in Chinese dental students and practicing dentists remain elusive.

The continuing dental education, in addition to predoctoral general dental education and postgraduate training, is another critical approach to improve dentists' knowledge and skills of diagnosing and treating TMD. Although some courses in TMD are available online or in-person, the content of these courses is highly variable. Not all recommendations of TMD treatment are evidence-based [7]. Up till now, few studies have focused on evaluating practicing dentists' knowledge in competencies of TMD and the efficacy of continuing education in China. Moreover, studies on TMD continuing education course design are also lacking.

The aim of this study is to evaluate the knowledge level and attitudes towards TMD among both postgraduate students and practicing dentists in western China. By comparing the differences of the two groups, we aim to reveal the current status of TMD education and provide our suggestions on predoctoral, postgraduate, and continuing education.

## 2. Materials and Methods

### 2.1. Participants

**Figure 1:**
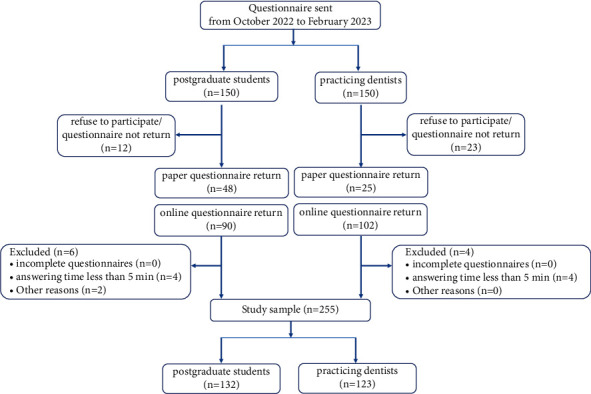
Study inclusion flow diagram.

The study was designed as an observational and descriptive cross-sectional study. Sampling was done by the convenience sampling method from October 1, 2022, to February 28, 2023. Questionnaires were distributed to 150 postgraduate dental students and 150 practicing dentists in paper form in public or private medical facility, postgraduate or practicing dentists training meetings in Chengdu, China. Participants were asked verbally whether they were postgraduate students or practicing dentists accredited by the Chinese National Health Commission before the questionnaire was delivered, and no personal identification was requested. The time to complete the questionnaire was recorded. At the same time, the questionnaire QR code is attached to the paper-based questionnaire, and the respondents can choose to complete the questionnaire by scanning the QR code in their free time, and the answering time was automatically detected. The deadline of online data collection was February 28, 2023. If respondents refused to participate or did not complete the questionnaire, they would not be contacted and reminded again. All participants took part voluntarily and received no financial compensation. The number of returned questionnaires was 265, giving a total response rate of 88% (Figure 1). The exclusion criteria were as follows: (1) participants refused to complete or did not return the questionnaire, (2) participants return the incomplete questionnaire, and (3) the time to complete the questionnaire was less than 5 minutes. The study was approved by the Research Ethics Committee from Sichuan University. Before answering the questionnaire, the participants provided informed consent.

### 2.2. Questionnaire

General information was collected in the first section of the survey, including the age and gender of participants, practicing specialty of the respondents, and whether they have taken any courses on TMD or occlusion.

Due to the lack of a widely accepted questionnaire in China, in the second part of the survey, a questionnaire modified and reorganized from published references was provided. At the same time, we exercised caution in the number of questions posed to prevent unsatisfactory data quality and limiting the number of items at 25. And almost all of the questions in the questionnaire have reached consensus among more than 75% of orofacial pain specialists or TMD specialists [6, 8, 9]. The questionnaire contains 25 items regarding attitudes towards 4 domains, chronic pain, etiology, diagnosis, treatment, and prognosis of TMD **(**Supplementary Table 1**)**. Items were answered on a 6-point scale, where 1 represents “strongly agree,” 2 represents “agree,” 3 represents “neutral,” 4 represents “disagree,” 5 represents “strongly disagree,” and 6 represents “I don't know.” “Group consensus” was defined as more than 50% of the respondents supported agree or disagree.

For dentists, we additionally asked about their years of practice, educational level (1: undergraduate, 2: master's degree, or 3: doctorate), type of medical facility (1: public or 2: private hospital), and responses to “What is your preferred treatment modality for patients with TMD?” (1: referral, 2: conservative treatment (including hot compress, physiotherapy, and pharmacological treatment), 3: orthodontic or prosthodontic treatment, or 4: splint therapy).

### 2.3. Statistical Analysis

The sample size was computed by using *G*∗power considering *α* (two-tailed) = 0.05 and power = 0.90. According to published articles [9], we assumed that if there is a different consensus on a statement between TMD experts and the control groups, the difference in their agreement is about 25% or more. The sample ratio (postgraduate dental students/practicing dentists) was determined to be 1 : 1. The analysis revealed that a total of 154 subjects were necessary to perform the study. Considering the 60% threshold response rate recommended for medical research, 300 subjects were finally included.

The data obtained from online or offline were exported at Excel software. Demographic information and percentages of the answer options in both groups were analyzed using SPSS software. We combined “strongly agree” and “agree” into “agree” and “strongly disagree” and “disagree” into “disagree” for the purpose of statistical analyses. Differences in answer options between the two groups for each statement were tested by the chi-square test. The difference was considered statistically significant when *P* < 0.05.

## 3. Results

A total of 132 postgraduate students and 123 dentists completed the questionnaires either online or offline. The postgraduate student group included 43 females (32.58%) and 89 males (67.42%). The dentist group included 31 females (25.20%) and 92 males (74.80%). The average age of participants in each group was 25.43 ± 1.92 and 35.46 ± 8.31 years, respectively. Around 75% of postgraduate students and 85% of dentists involved in the survey claimed that they have never taken any courses on TMD. Concerning education on occlusion, 30.30% of postgraduate students and 18.70% of dentists reported previously taking such courses **(**Table 1).

For the dentist group, the average years of practice of the dentists participating in this study were 12.42 ± 9.51 years, and most of the dentists had undergraduate education (72.36%) and worked in public hospitals (83.74%). In the process of clinical diagnosis and treatment, their preferred treatment modalities were conservative treatment (50.41%) and referral (38.21%) when patients presented TMD-related symptoms **(**Table 2**)**.

A total of 15 statements had statistically significant differences in responses between the postgraduate student and dentist groups (*P* < 0.05), and 9 of these statements had a different consensus. Practicing dentists generally agreed on “TMJ clicking is a serious symptom which often creates a painful condition,” “nocturnal bruxism is caused by occlusal interferences,” “reduced mouth opening capacity is almost never caused by TMJ arthritis,” “measuring mouth opening capacity is a reliable assessment method,” “orthodontic treatment can prevent the onset of TMD,” and “orthodontic treatment can treat TMD”, whereas students did not agree on these statements. Postgraduate students disagreed on “all individuals with TMJ clicking need treatment” and “TMD is more common amongst children with mixed dentition than amongst adult with permanent dentition,” and these two statements showed no consensus among practicing dentists. At the same time, the students and the dentists had different attitudes for the item 11, the former did not agree with the following: “the position of the condyle in the fossa as seen on tomogram is a very accurate indicator of internal derangement,” while the latter showed the opposite trend **(**Table 3**)**.

**Figure 2:**
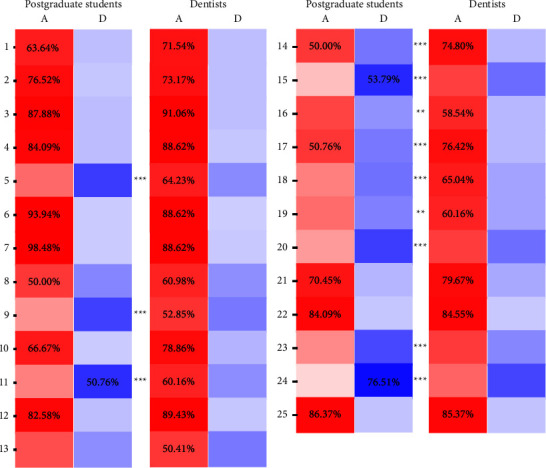
Distribution and consensus of agreement and disagreement between the postgraduate student and dentist groups for the 25 statements, and “neutral” and “I do not know” responses are not shown. Notes: agree and disagree with each statement are indicated using red and blue cells, respectively. The numbers in the cells indicate the respondents reached consensus on the statement, and the percentage of respondents who reached consensus. If there are no numbers in both red and blue cells for a statement, it indicates that the respondents have no consensus on the statement. Use an asterisk to indicate that there is a significant difference between the two groups regarding the statement, where ^*∗*^represents *P* < 0.05, ^*∗∗*^represents *P* < 0.01, and ^*∗∗∗*^represents *P* < 0.001. A: agree; D: disagree.

Further analysis demonstrated significant differences between the two groups in the proportion of agreeing on 12 statements (*P* < 0.05), even though both groups had the same consensus on some statements. The dentist group was more inclined to agree item 5, item 9, item 11, items 14 to 20, item 23, and item 24, while the attitude of the student group was opposite. These statements were in three domains, including etiology, diagnosis and treatment, and prognosis of TMD **(**Figure 2**)**.

We further analyzed the differences in responses between postgraduate students and practicing dentists in different subspecialties. The distribution of the answers to 6 items demonstrated differences between general dentistry and oral medicine departments. It is worth noting that for oral medicine specialists, there are differences in the responses of the following: “sleep disturbances are common in patients with chronic orofacial pain,” “depression can be an important etiologic factor in chronic orofacial pain,” and “counselling and behavioral therapy are the first line of treatment in patients which chronic TMD,” which is contrary to the results reported in Table 3. Compared to the postgraduate students, more practicing dentists chose to be neutral or opposed to these items. For prosthodontic specialists, no postgraduate students agreed with the following: “TMD is more common amongst children with mixed dentition than amongst adult with permanent dentition.” For orthodontists, postgraduate students were more likely to be neutral or disagree with the following: “the position of the condyle in the fossa as seen on tomogram is a very accurate indicator of internal derangement” and “orthodontic treatment can prevent the onset of TMD” than practicing dentists. Furthermore, postgraduate students in oral surgery were more likely to be neutral to the following: “depression can be an important etiologic factor in chronic orofacial pain” (Supplementary Table 2).

Comparing the answers of agreeing or disagreeing in different subspecialties, it is found that postgraduate students in general dentistry and orthodontics are more inclined to oppose items in diagnosis, treatment, and prognosis domains. Postgraduate students in oral medicine are more inclined to oppose statements in etiology, treatment, and prognosis domains. Postgraduate students in prosthodontics are more opposed to item 15, and postgraduate students in oral surgery are more opposed to item 5, compared with practicing dentists (Supplementary Table 3).

Finally, we analyzed differences in responses between dentists with different years of practice. We identified low practice year and high practice year groups based on the median years of practice (10 years). The results showed high agreement with almost all items between the two groups. And high practice year group demonstrated more agreement with the statement as follows: “TMJ clicking is a serious symptom which often creates a painful condition” and “patients with TMD who clench/brux do so either during the day or at night, but not both” **(**Supplementary Table 4).

## 4. Discussion

The purpose of this study is to investigate the differences of attitudes towards the etiology, diagnosis, and treatment of TMD between postgraduate students and practicing dentists. The analysis of current knowledge and attitudes of TMD management between these two groups might shed light on curriculum design improvement on campus and off campus in order to make dentists well prepared to deal with TMD.

Item 5 and item 24 discuss one of the common symptoms of TMD, TMJ clicking. 64.23% of the dentists agreed that it is “a serious symptom which often creates a painful condition,” but postgraduate students could not reach a consensus on this statement. More students tend to disagree with the statement that “all clicking TMJs need treatment.” According to the literature, TMJ clicking appears at early stages of disc displacement, which could be heard during mouth opening and/or mouth closing [10]. Patients with TMJ clicking do not necessarily feel pain or suffer limitation of jaw movement [11]. Therefore, not all TMJ clicking symptoms are serious and need treatment. More postgraduate students are opposed to this perception than practicing dentists, and their choices were more consistent with the newest evidence in this field. In addition, although both dentists with low and high practice years reach a consensus on item 5, dentists with high practice years are more inclined to agree that TMJ clicking could cause pain, which indicates that the update of TMD knowledge is also necessary among dentists with years of experience.

Item 7 evaluates the role of psychological factors in the development of TMD. Despite significantly more dentists held a neutral opinion, most dentists and postgraduate students agree that stress is an important risk factor associated with chronic TMD. Research Diagnostic Criteria for TMD (RDC/TMD) suggests that psychological status and psychosocial functions are important criteria for TMD diagnosis [12]. Great efforts have been made to emphasize the importance of psychological factors including stress, anxiety, and depression in chronic TMD development in dental education. This result indicates that both dentists and postgraduate students are well aware of this multifactorial nature of TMD.

Item 9 suggests the role of occlusal interference in nocturnal bruxism. In this study, 52.85% of the dentists showed their agreement, while most postgraduate students were neutral (28.79%) or disagreed (40.91%) with this. The idea that bruxism is caused by interference and malocclusion could be traced back to 1961 when Ramfjord and Ash concluded with a poor scientific method and no control group [13]. Up till now, there is no high-quality evidence of their relationships [14]. The exact cause of bruxism remains elusive. Practicing dentists have achieved consensus on this statement, and more dentists than students endorsed this statement. Dentists in this study seem to overlook the role of the occlusal factor in the onset of bruxism.

Although migraine occurs most commonly in the area of the ophthalmic branch of the trigeminal nerve, studies have found increased pain sensitivity of the masticatory muscles during migraine attacks [15, 16]. In turn, TMD also appear to be associated with headaches [17]. Due to the high prevalence of migraine and TMD in the population, it is meaningful to clarify the overlapping of signs and symptoms of migraine and TMD. There are more postgraduate students who are not clear about item 10, which indicates that they may lack knowledge and clinical experience in this area. But both postgraduate students and dentists showed a consensus on the relationship between migraine and facial pain.

Both dentists and postgraduate students agreed that occlusal grinding has been suggested as a useful early treatment modality for TMD, especially among practicing oral surgeons (74.29%) and general dentists (94.44%). However, according to the literature, there is no evidence that occlusal adjustments (grinding) are more or less effective than a placebo in TMD treatment [18]. Several clinical trials and systematic reviews have been focused on this topic and suggested that occlusal adjustment is not an evidence-supported practice and is restrictively recommended [19]. The choices of dentists and postgraduate students conflict with the newest knowledge on this topic.

The relationship between orthodontic treatment and TMD has also been a controversial topic for long. Our study reveals several significant differences in the attitude towards the role of orthodontic treatment in TMD between two groups. Regarding whether orthodontic treatment can prevent the onset of TMD (item 18), more than half of the dentists tended to the following: “strongly agree” or “agree” with the statement, while the percentage was only 26.52% among postgraduate students; 60.16% of the dentists “strongly agree” or “agree” with the idea that “orthodontic treatment can treat TMD” (item 19), whereas postgraduate students could not reach a consensus on this statement. As for whether “orthodontic therapy is the best treatment to resolve TMD in a patient with a skeletal malocclusion” (item 20), neither of the group showed consensus. Notably, there was no consensus on item 18 and item 20, even between orthodontists and postgraduate students in the department of orthodontics. Postgraduate students are more inclined to maintain a neutral attitude towards the following: “orthodontic treatment can prevent the onset of TMD” and express disapproval of the following: “orthodontic therapy is the best treatment to resolve TMD in a patient with a skeletal malocclusion.” However, half of the orthodontists agree that orthodontics plays a positive role in the prevention and treatment of TMD. Actually, since late 1990s, there have been multiple studies, including systematic reviews, suggesting that there is no support to believe that orthodontic treatment could prevent or treat TMD [20, 21].

Taken items 9, 17, 18, 19, and 20 together, the correlation between occlusal factors and TMD is still broadly supported, especially among practicing dentists. For subspecialties, oral medicine specialists and orthodontists were more likely to agree with the connection between the two. This result is consistent with previous studies conducted by De Medeiros Tormes et al. [4]. The worldwide false perception may be due to the fact that in many dental curricula, TMD is often taught in occlusion courses. Although recent studies have disproved their correlations [22, 23], stereotypes still persist as students and dentists are not updated with the newest evidence-based knowledge in time. The students' disagreement rates were 40.91%, 21.21%, 23.48%, 18.94%, and 42.42% in these items, suggesting their generally more prudent attitude towards the role of occlusion.

Item 11 addresses the use of radiology in the TMD diagnosis. While 60.16% of the dentists “strongly agree” or “agree” with the statement that the position of the condyle in the fossa on tomogram is a very accurate indicator of internal derangement, more postgraduate students show their neutral or opposing attitude towards this. The internal derangement of the articular disc is an abnormal positional relationship between the mandibular condyle and the articular eminence [24]. It is characterized by clicking in the affected joint or restricted movement. Of note, articular disc is not visualized on tomogram examinations. Therefore, in order to evaluate the disc position, magnetic resonance imaging, which shows the soft tissue components better, is often the modality of choice to provide definitive diagnosis [25]. It has been reported that internal derangement is correlated with alteration of condylar position in some cases. However, there may be limited translational motion of condyle when there is disc displacement without reduction [26]. It is inaccurate to claim that the position of condyle on tomogram is a very good indicator of internal derangement. This result demonstrates the lack of knowledge of choosing appropriate radiological approaches for the TMD diagnosis, especially for practicing dentists. About 60% of general dentists and 40% of orthodontists have inaccurate views. Inappropriate examination methods may underdiagnose or overdiagnose patients and make patients suffer unnecessary financial burden and radiation.

The significantly lower percentage of dentists selecting “I don't know” in each question suggests their more unwavering attitude towards the TMD diagnosis and treatment. However, as we discussed above, many of their opinions conflict with the newest evidence-based studies, which might lead to inappropriate decisions in clinical practice. The possible reason for the higher proportion of incorrect selections among practicing dentists might be the limited and outdated knowledge they get from general dental education since they have graduated for long. As many of them practice in private hospital, they barely have the opportunity to collaborate or communicate with professional TMD researchers or practitioners. The lack of access to peer reviewed scientific journals or other high-quality evidence in the field of TMD after they graduate is also an important barrier that keeps them from further studying. Postgraduate students, on the other hand, were more likely to select “I don't know” or “neutral” in each statement, which might suggest their hesitation and uncertainty towards TMD problems.

In the current study, a great majority dentists and postgraduate students have not taken any TMD courses. And some of their perspectives are inconsistent with the latest evidence-based knowledge. An important reason is the absence of specific TMD-related curriculum in dental education. As far as we know, there are few dental schools in China that offer professional TMD training courses. In most undergraduate dental schools in China, TMD is only mentioned in the curricula “oral and maxillofacial surgery” where no detailed clinical practice guidelines were provided. Dentists who practice right after graduating from college seldom have chances to get professional education concerning TMD treatment. For postgraduate dental education, TMD-related knowledge is taught in occlusion, prosthodontic, and orthodontic courses. However, none of these courses offers an overview of TMD multidisciplinary treatment based on the various factors involved in the diseases. Therefore, improving TMD education is essential to help dental students give patients better evidence-based care.

The significance of TMD and oral facial pain training has been realized recently all over the world. The American Dental Education Association states that dental student should have the capability to “prevent, diagnose, and manage temporomandibular disorders” [25]. In Sweden, students have access to exclusive TMD training program. Since 2020, Saudi Arabia has recognized oral facial pain as a distinct specialty in dentistry [6]. We suggest TMD instruction and assessment be solidly embedded in dental education programs. Curricular reform is also needed in China to reinforce the multidisciplinary TMD training using active learning methodologies in order to get students well prepared for TMD patients in future practice.

For predoctoral training, students should be introduced to basic techniques and methods for assessing patients with TMD under supervision and increasing their knowledge and interest in TMD. Students should be able to provide primary care for TMD patients and be aware of the necessity of further professional treatment. For postgraduate education, extensive knowledge is required including mastery of the integrated and multidimensional biopsychosocial model leading to TMD, accurate evidence for TMD diagnosis, and interdisciplinary management of TMD. TMD training courses should be separated from occlusion courses to prevent the false emphasis of occlusal factors in the onset of TMD. Postgraduate students should have skills in learning from literatures and assessing the limitations of published research. For continuing education, we suggest more courses either online or in-person could be provided from TMD specialists to practicing dentists. The courses should follow evidence-based guidelines and combine both theoretical and practical training, thus updating dentists with the newest knowledge and skills for the TMD diagnosis and treatment.

There are several strengths of this research. To the best of our knowledge, there are limited studies investigating the knowledge about TMD among dentists in China, especially in non-TMJ departments. Early identification and intervention of TMD patients should not be limited to TMJ departments. This study demonstrated that Chinese dentists have insufficient knowledge and awareness of TMD. Secondly, the questionnaire items in this study are based on published articles, so horizontal comparison can be made with the answers of experts and dentists in other countries, and it can provide a reference for the establishment of the Chinese version of the TMD knowledge questionnaire. Finally, this study surveyed postgraduate students and practicing doctors in different departments with a high response rate, which increased the generalizability of the study.

And the current study had some limitations. First, the validity of the questionnaire used in this study has not been verified, and a standardized and validated questionnaire would facilitate the comparison of conclusions between different studies. Second, the lack of repeated measurements limited the evaluation of the validity and reliability of the questionnaire. Third, insufficiencies in TMD knowledge among postgraduate students and practicing dentists were found in this study. In stratified analysis, some differences may not be statistically significant because of insufficient statistical power and limited sample size, and the extrapolation of conclusions is limited. Therefore, a larger sample size with subspecialty match might be required to conduct further stratified analysis. Fourth, the previous specialty of general dentists was not asked in this study. Since general dentists may have graduated from a particular specialty, there might be observational bias in the stratified analysis. In future studies, a more accurate analysis could be performed by surveying the type of subspecialty education received by practicing dentists.

## 5. Conclusions

This study suggests the lack of TMD training among practicing dentists and postgraduate students. Both groups exhibited insufficient knowledge and confidence in managing TMD problems. Therefore, curriculum reform is necessary in predoctoral, postgraduate, and continuing education to enhance their understanding and proficiency in diagnosing and treating TMD.

## Figures and Tables

**Table 1:** The demographic characteristics of the 255 participants included in the analysis.

	Postgraduate students	Dentists
*N*	132	123
Age, mean ± SD	25.43 ± 1.92	35.46 ± 8.31
Gender, *n* (%)		
Male	89 (67.42%)	92 (74.80%)
Female	43 (32.58%)	31 (25.20%)
Department, *n* (%)		
General dentistry	6 (4.54%)	66 (53.66%)
Oral medicine	33 (25.00%)	20 (16.26%)
Prosthodontics	30 (22.73%)	5 (4.07%)
Oral surgery	16 (12.12%)	13 (10.57%)
Orthodontics	41 (31.06%)	7 (5.69%)
TMJ	1 (0.76%)	1 (0.81%)
Two or more departments	5 (3.79%)	11 (8.94%)
TMD education, *n* (%)	32 (24.24%)	18 (14.63%)
Occlusion education, *n* (%)	40 (30.30%)	23 (18.70%)

SD: standard deviation; TMJ: temporomandibular joint; TMD: temporomandibular disorders.

**Table 2:** Additional demographic information of the dentists.

	Dentists	
	Mean or frequency	SD or percentage
Years of practice	12.42	9.51
Educational level		
Undergraduate	89	72.36
Master's degree	23	18.70
Doctorate	11	8.94
Type of medical facility		
Public	103	83.74
Private	20	16.26
Preferred treatment modality		
Referral	47	38.21
Conservative treatment	62	50.41
Orthodontic/prosthodontic treatment	10	8.13
Splint therapy	4	3.25

SD: standard deviation.

**Table 3:** Comparison between postgraduate students and practicing dentists answer options to different statements.

Item	Postgraduate students					Dentists					*P*
	Strongly agree and agree	Neutral	Strongly disagree and disagree	I do not know	Consensus	Strongly agree and agree	Neutral	Strongly disagree and disagree	I do not know	Consensus	
1	84 (63.64%)	31 (23.48%)	4 (3.03%)	13 (9.85%)	A	88 (71.54%)	26 (21.14%)	4 (3.25%)	5 (4.07%)	A	0.289
2	101 (76.52%)	21 (15.91%)	2 (1.52%)	8 (6.05%)	A	90 (73.17%)	27 (21.95%)	4 (3.25%)	2 (1.63%)	A	0.147
3	116 (87.88%)	11 (8.33%)	5 (3.79%)	0 (0%)	A	112 (91.06%)	6 (4.88%)	4 (3.25%)	1 (0.81%)	A	0.534
4	111 (84.09%)	13 (9.85%)	5 (3.79%)	3 (2.27%)	A	109 (88.62%)	12 (9.76%)	2 (1.63%)	0 (0%)	A	0.322
5	43 (32.58%)^a^	29 (21.97%)^a,b^	57 (43.18%)^b^	3 (2.27%)^a,b^	N	79 (64.23%)^a^	22 (17.89%)^a,b^	20 (16.26%)^b^	2 (1.63%)^a,b^	A	<0.001 ^*∗∗∗*^
6	124 (93.94%)	5 (3.79%)	1 (0.76%)	2 (1.51%)	A	109 (88.62%)	13 (10.57%)	0 (0%)	1 (0.81%)	A	0.072
7	130 (98.48%)^a^	1 (0.76%)^b^	1 (0.76%)^a,b^	0 (0%)^a,b^	A	109 (88.62%)^a^	12 (9.76%)^b^	1 (0.81%)^a,b^	1 (0.81%)^a,b^	A	0.001 ^*∗∗*^
8	66 (50.00%)	30 (22.73%)	23 (17.42%)	13 (9.85%)	A	75 (60.98%)	25 (20.33%)	18 (14.63%)	5 (4.07%)	A	0.185
9	29 (21.97%)^a^	38 (28.79%)^b^	54 (40.91%)^b^	11 (8.33%)^b^	N	65 (52.85%)^a^	30 (24.39%)^b^	26 (21.13%)^b^	2 (1.63%)^b^	A	<0.001 ^*∗∗∗*^
10	88 (66.67%)^a^	24 (18.18%)^a,b^	1 (0.76%)^a^	19 (14.39%)^b^	A	97 (78.86%)^a^	16 (13.01%)^a,b^	7 (5.69%)^a^	3 (2.44%)^b^	A	<0.001 ^*∗∗∗*^
11	35 (26.52%)^a^	22 (16.67%)^a,b^	67 (50.76%)^c^	8 (6.05%)^b,c^	D	74 (60.16%)^a^	28 (22.76%)^a,b^	19 (15.45%)^c^	2 (1.63%)^b,c^	A	<0.001 ^*∗∗∗*^
12	109 (82.58%)	13 (9.85%)	4 (3.03%)	6 (4.54%)	A	110 (89.43%)	10 (8.13%)	2 (1.63%)	1 (0.81%)	A	0.232
13	55 (41.67%)^a^	37 (28.03%)^a,b^	20 (15.15%)^a^	20 (15.15%)^b^	N	62 (50.41%)^a^	31 (25.2%)^a,b^	26 (21.13%)^a^	4 (3.25%)^b^	A	0.006 ^*∗∗*^
14	66 (50.00%)^a^	31 (23.48%)^a,b^	29 (21.97%)^b^	6 (4.55%)^a,b^	A	92 (74.80%)^a^	24 (19.51%)^a,b^	6 (4.88%)^b^	1 (0.81%)^a,b^	A	<0.001 ^*∗∗∗*^
15	16 (12.12%)^a^	25 (18.94%)^b^	71 (53.79%)^c^	20 (15.15%)^b,c^	D	57 (46.34%)^a^	29 (23.58%)^b^	31 (25.20%)^c^	6 (4.88%)^b,c^	N	<0.001 ^*∗∗∗*^
16	60 (45.45%)^a^	51 (38.64%)^a,b^	19 (14.39%)^b^	2 (1.52%)^a,b^	N	72 (58.54%)^a^	43 (34.96%)^a,b^	6 (4.88%)^b^	2 (1.63%)^a,b^	A	0.029 ^*∗*^
17	67 (50.76%)^a^	28 (21.21%)^a,b^	28 (21.21%)^b^	9 (6.82%)^b^	A	94 (76.42%)^a^	22 (17.89%)^a,b^	6 (4.88%)^b^	1 (0.81%)^b^	A	<0.001 ^*∗∗∗*^
18	35 (26.52%)^a^	59 (44.7%)^b^	31 (23.48%)^b^	7 (5.30%)^b^	N	80 (65.04%)^a^	31 (25.20%)^b^	12 (9.76%)^b^	0 (0%)^b^	A	<0.001 ^*∗∗∗*^
19	42 (31.82%)^a^	59 (44.7%)^b^	25 (18.94%)^b^	6 (4.54%)^a,b^	N	74 (60.16%)^a^	36 (29.27%)^b^	12 (9.76%)^b^	1 (0.81%)^a,b^	A	<0.001 ^*∗∗∗*^
20	26 (19.70%)^a^	38 (28.79%)^b^	56 (42.42%)^b^	12 (9.09%)^b^	N	59 (47.97%)^a^	31 (25.20%)^b^	30 (24.39%)^b^	3 (2.44%)^b^	N	<0.001 ^*∗∗∗*^
21	93 (70.45%)	21 (15.92%)	7 (5.30%)	11 (8.33%)	A	98 (79.67%)	12 (9.76%)	10 (8.13%)	3 (2.44%)	A	0.062
22	111 (84.09%)	14 (10.61%)	2 (1.52%)	5 (3.78%)	A	104 (84.55%)	16 (13.01%)	1 (0.81%)	2 (1.63%)	A	0.653
23	32 (24.24%)^a^	39 (29.55%)^a,b^	50 (37.88%)^b^	11 (8.33%)^b^	N	60 (48.78%)^a^	39 (31.71%)^a,b^	21 (17.07%)^b^	3 (2.44%)^b^	N	<0.001 ^*∗∗∗*^
24	10 (7.58%)^a^	17 (12.88%)^a,b^	101 (76.51%)^c^	4 (3.03%)^b,c^	D	42 (34.15%)^a^	33 (26.83%)^a,b^	48 (39.02%)^c^	0 (0%)^b,c^	N	<0.001 ^*∗∗∗*^
25	114 (86.37%)	11 (8.33%)	3 (2.27%)	4 (3.03%)	A	105 (85.37%)	14 (11.38%)	3 (2.44%)	1 (0.81%)	A	0.573

*Notes*. Data are expressed as *N* (percentage). Different superscript letters indicate significant differences; without superscript or marked with the same letter represent no significant differences. ^*∗*^*P* < 0.05, ^*∗∗*^*P* < 0.01, and ^*∗∗∗*^*P* < 0.001. A: agree; N: no consensus; D: disagree.

## Data Availability

The data used to support the findings of this study are available from the corresponding author upon request.

## Supplementary Materials

Supplementary Materials Supplementary Table 1: twenty-five knowledge statements in four domains. Supplementary Table 2: comparison between postgraduate students and practicing dentists answer options in different subspecialties, and only statistically significant results are presented. Supplementary Table 3: distribution of agreement and disagreement between the postgraduate student and dentist groups in different subspecialties, and only statistically significant results are presented. Supplementary Table 4: comparison between high years of practice and low years of practice groups answer options in practicing dentists, and only statistically significant results are presented.
