# Supplementary material for: Knowledge and Attitudes regarding Temporomandibular Disorders among Postgraduate Dental Students and Practicing Dentists in Western China: A Questionnaire-Based Observational Investigation
Source: Pain Res Manag. 2023 Jul 18;2023:7886248. doi: 10.1155/2023/7886248 (PMC10368496; doi:10.1155/2023/7886248)
Supplement: Supplementary Materials — Supplementary Table 1: twenty-five knowledge statements in four domains. Supplementary Table 2: comparison between postgraduate students and practicing dentists answer options in different subspecialties, and only statistically significant results are presented. Supplementary Table 3: distribution of agreement and disagreement between the postgraduate student and dentist groups in different subspecialties, and only statistically significant results are presented. Supplementary Table 4: comparison between high years of practice and low years of practice groups answer options in practicing dentists, and only statistically significant results are presented. [file 7886248.f1.docx]

**Supplement table 1. Twenty five knowledge statements in four domains**

| **Domain** | **Item** | **Statement** |
| --- | --- | --- |
| **TMD-related pain** | 1 | - Sleep disturbances are common in patients with chronic orofacial pain. |
|  | 2 | - Depression can be an important etiologic factor in chronic orofacial pain. |
|  | 3 | - Pain is the most common reason to seek treatment of TMD. |
|  | 4 | - TMD pain is aggravated/relieved by jaw motion. |
| **Etiology** | 5 | - TMJ clicking is a serious symptom which often creates a painful condition. |
|  | 6 | - Oral parafunctional habits are often significant in the development of chronic TMD. |
|  | 7 | - Stress is a very important factor in the development of chronic TMD. |
|  | 8 | - Patients with TMD who clench/brux do so either during the day or at night, but not both. |
|  | 9 | - Nocturnal bruxism is caused by occlusal interferences. |
|  | 10 | - Migraine can cause or is comorbid with facial/ jaw pain. |
| **Diagnosis** | 11 | - The position of the condyle in the fossa as seen on tomogram is a very accurate indicator of internal derangement |
|  | 12 | - Examination of neck muscles and TMJ with patients with orofacial chronic pain is important. |
|  | 13 | - Reduced mouth opening capacity is almost never caused by TMJ arthritis. |
|  | 14 | - Palpatory tenderness in the masticatory system and/or TMJ is the most important clinical sign of TMD. |
|  | 15 | - TMD is more common amongst children with mixed dentition than amongst adult with permanent dentition. |
|  | 16 | - Measuring mouth opening capacity is a reliable assessment method. |
| **Treatment** **and prognosis** | 17 | - Occlusal grinding is a useful early treatment modality for TMD. |
|  | 18 | - Orthodontic treatment can prevent the onset of TMD. |
|  | 19 | - Orthodontic treatment can treat TMD. |
|  | 20 | - Orthodontic therapy is the best treatment to resolve TMD in a patient with a skeletal malocclusion. |
|  | 21 | - Anti-inflammatory drugs are effective in the treatment of acute arthralgia. |
|  | 22 | - The use of an occlusal splint is a good therapy in patients with TMD. |
|  | 23 | - Occlusal splints can eliminate bruxism. |
|  | 24 | - All individuals with TMJ clicking need treatment. |
|  | 25 | - Counselling and behavioral therapy are the first line of treatment in patients which chronic TMD. |

Abbreviation: TMJ: temporomandibular joint, TMD: temporomandibular disorders.

**Supplement table 2.** **Comparison between postgraduate students and practicing dentists answer options in different subspecialties, and only statistically significant results are presented**

|  | **Postgraduate students** | |  |  |  | **Dentists** | |  |  |  |  |
| --- | --- | --- | --- | --- | --- | --- | --- | --- | --- | --- | --- |
| Item | Strongly agree and agree | Neutral | Strongly disagree and disagree | I don’t know | Consensus | Strongly agree and agree | Neutral | Strongly disagree and disagree | I don’t know | Consensus | P |
| General dentistry |  |  |  |  |  |  |  |  |  |  |  |
| 14 | 3 (33.33%) | 2 (22.22%) | 3 (33.33%) | 1 (11.11%) | N | 55 (78.57%) | 15 (21.43%) | 0 (0%) | 0 (0%) | A | **<0.001***** |
| 15 | 1 (11.11%) | 4 (44.44%) | 2 (22.22%) | 2 (22.22%) | N | 38 (54.29%) | 18 (25.71%) | 11 (15.71%) | 3 (4.29%) | A | **0.023*** |
| 17 | 6 (66.67%) | 1 (11.11%) | 1 (11.11%) | 1 (11.11%) | A | 52 (74.29%) | 17 (24.29%) | 1 (1.43%) | 0 (0%) | A | **0.047*** |
| 23 | 2 (22.22%) | 2 (22.22%) | 4 (44.44%) | 1 (11.11%) | N | 38 (54.29%) | 22 (31.43%) | 9 (12.86%) | 1 (1.43%) | A | **0.029*** |
| 24 | 0 (0%) | 3 (33.33%) | 6 (66.67%) | 0 (0%) | D | 26 (37.14%) | 23 (32.86%) | 21 (30.00%) | 0 (0%) | N | **0.028*** |
| Oral medicine |  |  |  |  |  |  |  |  |  |  |  |
| 1 | 20 (58.82%) | 6 (17.65%) | 0 (0%) | 8 (23.53%) | A | 17 (65.38%) | 9 (34.62%) | 0 (0%) | 0 (0%) | A | **0.008**** |
| 2 | 27 (79.41%) | 4 (11.76%) | 0 (0%) | 3 (8.82%) | A | 16 (61.54%) | 9 (34.62%) | 1 (3.85%) | 0 (0%) | A | **0.022*** |
| 5 | 9 (26.47%) | 9 (26.47%) | 15 (44.12%) | 1 (2.94%) | N | 16 (61.54%) | 2 (7.69%) | 7 (26.92%) | 1 (3.85%) | A | **0.026*** |
| 18 | 8 (23.53%) | 13 (38.24%) | 10 (29.41%) | 3 (8.82%) | N | 15 (57.69%) | 8 (30.77%) | 3 (11.54%) | 0 (0%) | A | **0.019*** |
| 19 | 8 (23.53%) | 16 (47.06%) | 9 (26.47%) | 1 (2.94%) | N | 16 (61.54%) | 7 (26.92%) | 3 (11.54%) | 0 (0%) | A | **0.018*** |
| 25 | 32 (94.12%) | 0 (0%) | 0 (0%) | 2 (5.88%) | A | 22 (84.62%) | 3 (11.54%) | 1 (3.85%) | 0 (0%) | A | **0.023*** |
| Prosthodontics |  |  |  |  |  |  |  |  |  |  |  |
| 15 | 0 (0%) | 10 (29.41%) | 13 (38.24%) | 11 (32.35%) | N | 4 (33.33%) | 0 (0%) | 7 (58.33%) | 1 (8.33%) | D | **<0.001***** |
| Oral surgery |  |  |  |  |  |  |  |  |  |  |  |
| 2 | 10 (58.82%) | 6 (35.29%) | 0 (0%) | 1 (5.88%) | A | 15 (83.33%) | 1 (5.56%) | 2 (11.11%) | 0 (0%) | A | **0.026*** |
| 5 | 3 (17.65%) | 4 (23.53%) | 10 (58.82%) | 0 (0%) | D | 12 (66.67%) | 3 (16.67%) | 3 (16.67%) | 0 (0%) | A | **0.014*** |
| 17 | 8 (47.06%) | 3 (17.65%) | 3 (17.65%) | 3 (17.65%) | N | 17 (94.44%) | 0 (0%) | 1 (5.56%) | 0 (0%) | A | **0.008**** |
| Orthodontics |  |  |  |  |  |  |  |  |  |  |  |
| 11 | 6 (13.95%) | 5 (11.63%) | 31 (72.09%) | 1 (2.33%) | D | 6 (42.86%) | 3 (21.43%) | 5 (35.71%) | 0 (0%) | N | **0.038*** |
| 18 | 8 (18.6%) | 23 (53.49%) | 12 (27.91%) | 0 (0%) | N | 7 (50.00%) | 3 (21.43%) | 4 (28.57%) | 0 (0%) | A | **0.045*** |
| TMJ |  |  |  |  |  |  |  |  |  |  |  |
| NS |  |  |  |  |  |  |  |  |  |  |  |

Notes: Data are expressed as N (percentage). * P<0.05, ** P<0.01, *** P<0.001.

Abbreviation: A: agree, N: no consensus, D: disagree, TMJ: temporomandibular joint, NS: no significant difference.

**Supplement table 3.** **Distribution of agreement and disagreement between the postgraduate student and dentist groups in different subspecialties, and only statistically significant results are presented**

|  | **Postgraduate students** | | **Dentists** | |  |
| --- | --- | --- | --- | --- | --- |
| Item | Strongly agree and agree | Strongly disagree  and disagree | Strongly agree  and agree | Strongly disagree  and disagree | P |
| General dentistry |  |  |  |  |  |
| 11 | 4 (44.44%) | 3 (33.33%) | 48 (68.57%) | 5 (7.14%) | **0.043*** |
| 14 | 3 (33.33%) | 3 (33.33%) | 55 (78.57%) | 0 (0%) | **0.001**** |
| 16 | 3 (33.33%) | 2 (22.22%) | 42 (60%) | 1 (1.43%) | **0.025*** |
| 23 | 2 (22.22%) | 4 (44.44%) | 38 (54.29%) | 9 (12.86%) | **0.027*** |
| 24 | 0 (0%) | 6 (66.67%) | 26 (37.14%) | 21 (30%) | **0.023*** |
| Oral medicine |  |  |  |  |  |
| 5 | 9 (26.47%) | 15 (44.12%) | 16 (61.54%) | 7 (26.92%) | **0.041*** |
| 18 | 8 (23.53%) | 10 (29.41%) | 15 (57.69%) | 3 (11.54%) | **0.035*** |
| 19 | 8 (23.53%) | 9 (26.47%) | 16 (61.54%) | 3 (11.54%) | **0.033*** |
| 23 | 5 (14.71%) | 15 (44.12%) | 11 (42.31%) | 7 (26.92%) | **0.047*** |
| Prosthodontics |  |  |  |  |  |
| 15 | 0 (0%) | 13 (38.24%) | 4 (33.33%) | 7 (58.33%) | **0.031*** |
| Oral surgery |  |  |  |  |  |
| 5 | 3 (17.65%) | 10 (58.82%) | 12 (66.67%) | 3 (16.67%) | **0.007**** |
| Orthodontics |  |  |  |  |  |
| 11 | 6 (13.95%) | 31 (72.09%) | 6 (42.86%) | 5 (35.71%) | **0.044*** |
| 20 | 6 (13.95%) | 24 (55.81%) | 7 (50%) | 4 (28.57%) | **0.019*** |
| TMJ |  |  |  |  |  |
| NS |  |  |  |  |  |

Notes: Data are expressed as N (percentage). * P<0.05, ** P<0.01, *** P<0.001.

Abbreviation: TMJ: temporomandibular joint, NS: no significant difference.

**Supplement table 4.** **Comparison between high years of practice and low years of practice groups answer options in practicing dentists, and only statistically significant results are presented**

| Item | Group | Strongly agree and agree | Neutral | Strongly disagree and disagree | I don’t know | Consensus | P |
| --- | --- | --- | --- | --- | --- | --- | --- |
| 5 | Low practice year | 35 (54.68%) | 12 (18.75%) | 15 (23.44%) | 2 (3.13%) | A | **0.037*** |
|  | High practice year | 44 (74.58%) | 10 (16.95%) | 5 (8.47%) | 0 (0%) | A |  |
| 8 | Low practice year | 32 (50.00%) | 14 (21.88%) | 13 (20.31%) | 5 (7.81%) | A | **0.008**** |
|  | High practice year | 43 (72.88%) | 11 (18.65%) | 5 (8.47%) | 0 (0%) | A |  |

Notes: Data are expressed as N (percentage). * P<0.05, ** P<0.01, *** P<0.001.

Abbreviation: A: agree.
